# Receptivity to malaria: meaning and measurement

**DOI:** 10.1186/s12936-022-04155-0

**Published:** 2022-05-08

**Authors:** Joshua O. Yukich, Kim Lindblade, Jan Kolaczinski

**Affiliations:** 1 Department of Tropical Medicine, Center for Applied Malaria Research and Evaluation, Tulane University School of Public Health and Tropical Medicine, New Orleans, LA USA; 2 Global Malaria Programme, World Health Organization, Geneva, CH USA

**Keywords:** Malaria transmission measurement, Elimination, Receptivity, Transmission networks

## Abstract

“Receptivity” to malaria is a construct developed during the Global Malaria Eradication Programme (GMEP) era. It has been defined in varied ways and no consistent, quantitative definition has emerged over the intervening decades. Despite the lack of consistency in defining this construct, the idea that some areas are more likely to sustain malaria transmission than others has remained important in decision-making in malaria control, planning for malaria elimination and guiding activities during the prevention of re-establishment (POR) period. This manuscript examines current advances in methods of measurement. In the context of a decades long decline in global malaria transmission and an increasing number of countries seeking to eliminate malaria, understanding and measuring malaria receptivity has acquired new relevance.

## Main Text

“Receptivity” to malaria is a construct which was developed during the Global Malaria Eradication Programme (GMEP) era [1]. Its meaning is commonly accepted as the degree to which a certain place supports local malaria transmission. This work reported in this manuscript was undertaken to support the World Health Organization in updating the definitions of and guiding the utilization of this construct for malaria elimination and the prevention of re-establishment of malaria. The formal definition of “receptivity” as presented in the most current update of the WHO malaria terminology [2] is:

[The] degree to which an ecosystem in a given area at a given time allows for the transmission of *Plasmodium* spp. from a human through a vector mosquito to another human.

*Note*: This concept reflects vectorial capacity, susceptibility of the human population to malaria infection, and the strength of the health system, including malaria interventions. Receptivity depends on vector susceptibility to particular species of *Plasmodium*, and is influenced by ecological and climatic factors.

The footnotes to the definition of “receptivity” make it clear that receptivity is a property of an ecosystem, and that this ecosystem is implied to contain humans, vectors and climatic factors, each of which must also possess the qualities of susceptibility, competence, and suitability, respectively [3].

The construct or “receptivity” itself is a critical component necessary for malaria elimination or prevention of reintroduction programmes, to plan and stratify their programme areas. Despite the importance of understanding and measuring “receptivity” for these purposes there is very little directive guidance available to programme and policy-makers on how to do so. This review describes the results of a narrative review of the literature on malaria “receptivity” with the main aim of providing context and guidance around measurement and use of receptivity as a practical, measurable and useful construct for malaria elimination and prevention of re-establishment planning and evaluation. Ultimately the findings of the review suggest that there may not be one standard metric for “receptivity” that will be measurable, easily available and practical in all locations, but that a number of approaches to doing so have been utilized which programmes should consider for use. The World Health Organization (WHO) *Framework for Malaria Elimination* provides a framework for the practical use of the concept in stratification, but provides little guidance on how to measure or assess the construct [4].

The WHO standard definition of receptivity, even after revision, does not explicitly provide a direct and single standard measurement tool for the construct. Additional guidance on how to assess, measure or estimate the level of “receptivity” for a given place and time is necessary. A set of tools and guidance on measurement of “receptivity” is necessary for programmes to effectively use this construct in malaria risk stratification, tailoring intervention strategies or surveillance. This manuscript reviews the history and current use of this term in the scientific and gray literature as it pertains to malaria transmission, with the aim of providing an updated definition of the term “receptivity” now included in the WHO’s Malaria Terminology [2]) and exploration of new advances in the measurement and estimation of receptivity, which have most recently become available to malaria control and elimination programmes. The strategies outlined here, can hopefully be integrated by programmes undergoing stratification or planning elimination and prevention of re-establishment programmes especially as outlined through the Framework for malaria elimination [4].

### Historical descriptions of receptivity

A huge fraction of published studies that deal with issues of malaria receptivity fail to provide any qualitative description of the term despite deploying it. Most studies provide no quantitative definition of the term at all even when they rely on the concept heavily in their discussion (or even sometime in the manuscript titles). The most extensive description in the literature comes from a WHO document published in 2014 [5]. That description is as follows:

Every place in the world has a certain potential for malaria transmission that is intrinsic to it at a given point in time, ranging from zero to some level above zero. This characteristic is often referred to as ‘receptivity,’ and indicates the extent to which conditions are favourable for malaria transmission in a specific location. The potential for malaria transmission is a function of many varied factors, including (but not limited to):

- The mosquito vector species, their abundance and behaviour
- The *Plasmodium* species
- Temperature and rainfall
- Geography and topography of the land
- Amount and type of agriculture or land-cover in that area
- Strength of the health system
- Quality of housing in which people live
- How people spend their time in the places and times when vectors are feeding.

Together, these characteristics will lead to a specific malaria baseline: the level of malaria burden that would exist in a given place if no interventions are implemented to control it.

This description made clear that, at least as used in some places, measurement of receptivity by assessment of its contributing factors would be a complex process which would require measurement of factors across a number of domains, requiring differing areas of expertise. It also highlights a separate issue, which is that “receptivity” pertains to a specific time and place as does the WHO standard definition [3]. Therefore, any implementable use of this construct needs to decide if the construct applies to the current state of the system, a historical state of the system or a current counter-factual state of the system and develop a relevant operationalization of “place.” The WHO *Framework for Malaria Elimination* formalizes place as the smallest geographical unit for which implementation decisions can be made [4]. While most qualitative definitions used in the literature appear at face value to be agnostic to the specification of the time and place components of “receptivity.”

Other descriptions related “receptivity” to the “outbreak risk”, “natural endemic level,” or “intrinsic transmission potential,” referring not to only vector specific properties, but rather to joint vector and human epidemiological properties. These descriptions seek to use properties of infection incidence (Force of Infection) or possibly parasite prevalence - elements more directly related to transmission—as the defining characteristic of “receptivity.”

### Quantitative definitions of receptivity

The quantitative definitions of “receptivity” used in the literature are also varied. There are a minimum of five distinct quantitative definitions given or used in the literature. These include vectorial capacity (per Macdonald or Garrett-Jones), vector competence, the basic reproductive number, the reproductive number under control, the effective reproductive number, spleen rate or parasite prevalence among 2–9 year old children, having more than a minimum threshold density of competent vectors, high larval density per breeding site, historical prevalence data or modeled counter-factual estimates thereof, and maximum historical *Plasmodium falciparum* Parasite Rate in 2–10 year old children (*Pf*PR$_{2-10}$) (*Pv*PR$_{1-99}$ can be considered an analogous age standardized measure of parasite prevalence for *Plasmodium vivax* malaria and should be considered an alternative throughout this manuscript where *Pf*PR$_{2-10}$ is used). These definitions raise many of the same questions that arise when reviewing the qualitative descriptions used.

### Vectorial capacity

The most common quantitative definition of receptivity in the literature reviewed is “vectorial capacity [6–15].” This is also generally described as a mathematical function of several entomological properties and was derived by Garrett-Jones based on the Ross-Macdonald model [16]. The classical formulation of this indicator of “receptivity” follows Eq. 1:

1 $$\begin{aligned} C = \frac{ma^{2}p^{n}}{-ln(p)} \end{aligned}$$

where *C* is vectorial capacity or “receptivity”, *m* is mosquito density (per human), *ma* is the human biting rate—defined as the number of bites per person per day, *a* is the human-biting habit (which is defined as the expected number of bites on humans of one mosquito per day), *p* is the proportion of mosquitoes surviving one day (or the probability that a single mosquito survives a single day), and *n* is the length in days of the extrinsic incubation period, which is usually estimated based on the methods outlined in Detinova 1962 [17]. This equation is meant to express the number of new infections arising per day from all the bites on one infectious human assuming that all bites on the human lead to successful infection of the mosquito (and that all humans are completely susceptible as well). As such it cannot be interpreted without further consideration of vector competence for the proposed vector-parasite pairing.

The vectorial capacity model of “receptivity” was intended to capture all of the purely entomological components of the basic reproductive number for malaria. Some components are, however, challenging or impossible to directly measure. These include *a*, the (hu)man biting habit (which in field application is measured by the human blood index (HBI) as proxy - HBI is the proportion of blood fed mosquitoes whose mid-gut contents react to $\alpha$-human IgG in an ELISA assay or show evidence of human DNA by PCR after DNA extraction from the mosquito abdomen), and *p* which is also measured by a proxy known as the parous rate (*p* is estimated by assuming a stable mosquito population size and age structure and then estimated based on the measurement of the proportion of female mosquitoes caught, usually using a CDC light trap, which have ever laid eggs).

Vectorial capacity has been updated over time in theoretical work to include aspects of mosquito larval ecology and heterogeneity [18, 19]. Though in all the field studies identified in which vectorial capacity was estimated based on measured entomological data, the classical formulation, as presented here, was used [6–15].

Vectorial capacity as a proxy for the “receptivity” of an area poses measurement challenges mainly because measuring the component entomological elements require different sampling methods, which are rarely carried out at the same time and location. Even when they are jointly conducted, they may suffer from extreme local spatial and temporal variation and limits to the precision of measurement. Extrapolation of values from one site to a nearby location may result in significant bias or imprecision, though the use of model based geo-statistics might potentially help programs to interpolate sparse vectorial capacity data. Proxies for vectorial capacity which rely on simpler constructions such as biting rates alone have been suggested [20]. Biting rates alone have been suggested to serve as sufficient proxies to detect the most important components of spatial and temporal variation in *Anopheline* exposure [21]. Recent work has also suggested that exposure to whole *Anopheline* saliva can leave detectable immunological signals that may also be able to resolve the important differences in biting exposure and receptivity between locations [22].

Vectorial capacity could play a key role in stratification of areas, but its application will be limited by the serious measurement challenges associated with capturing each measure, statistical challenges in extrapolation between times and places, as well as a lack of robust historical databases, and repeated measurements over time. Operationalizing the measurement of “receptivity” through the measurement of vectorial capacity may be most useful where, vectorial capacity and hence “receptivity” can be shown to be absent due to a lack of the presence of suitable malaria vectors.

### The reproductive numbers

The other main group of quantitative definitions identified in the literature focused on some version of the reproductive number: $R_{0}, R_{C}$, or $R_{e}$. Reproductive numbers are meant to express the relative reproduction rate of a single infection (usually in terms of number of new human infections produced under varying circumstances) [23–29]. $R_{0}$, or the Basic Reproductive Number, is the classic example of a reproductive number in mathematical epidemiology and is meant to express the maximum transmission potential of a newly introduced infection in a completely naive population in the absence of any interventions to reduce or prevent disease spread. It is technically a ratio, but can also be thought of as a rate expressed in parasite generational time. Because of the long and variable generational interval (with many secondary infections arising 100-200 days or potentially longer after primary infections) for malaria, especially when untreated, very high values of $R_{0}$ can arise where transmission potential is high and treatment is uncommon [24, 30–32]. The generational interval for an infectious disease is the average time from the start of one infection to the start of a secondary infection caused by the primary infection, while the serial interval is the average time from the start of symptoms or detection of a disease to the detection of a secondary case of disease caused by the primary case. In the context of malaria these both refer to the time between human infections or human cases of disease.

The basic reproductive number only corresponds to the very initial rate of increase in an epidemic as very rapidly elements such as acquired immunity begin to act to slow the rate of increase. The basic reproductive number probably only realistically reflects the state of a population that is far enough into a stage of prevention of re-establishment that acquired immunity in the population has waned due to the birth of susceptible cohorts or the length of time since any malaria exposure occurred in the population.

An analogous construct is the controlled reproductive number, $R_{C}$. This ratio (or rate, similarly to $R_{0}$) expresses the maximum transmission potential in a situation with control interventions and treatment in place. Similar to $R_{0}$, this number is meant to express the number of human infections arising from one introduced human infection in one parasite generation. Also similar to $R_{0}$ the actual rate of reproduction only occurs during the very initial stages of introduction of malaria as further immune acquisition and other feedback mechanisms will rapidly alter parasite reproduction rates.

**Fig. 1:**
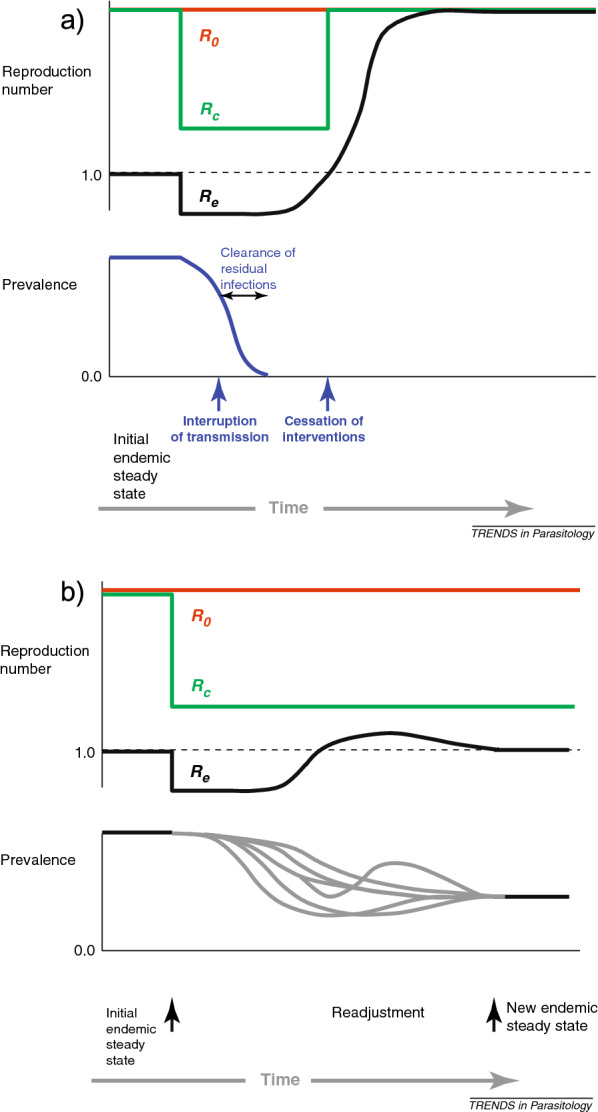
These figures, originally published in [54], illustrate potential relationships between $R_{0}$, $R_{C}$, and $R_{e}$ in the context where elimination is achieved (a) or a new lower endemic equilibrium is reached (b) by the imposition of control measures

Both $R_{0}$ and $R_{C}$ should be distinguished from the effective reproductive number, $R_{e}$, which is simply the ratio of new human infections arising per current human infection at any given point in time. The relationships between these three numbers is illustrated in Figure 1. It may be at first confusing that $R_{e}$ never achieves the level of $R_{C}$ or $R_{0}$ except after elimination. This occurs because these reproductive ratios cannot be achieved practically except at the initial stage of parasite invasion into a community without any other infected humans.

It is commonly understood that values of $R_0\ge 1$ correspond to situations in which an invading pathogen will establish endemicity, and values of $R_0<1$ correspond to situations in which an invading pathogen will fail to establish endemicity, and values of $0<R_{0}<1$ correspond to situations in which an invading pathogen might establish short chains of transmission that eventually end and fail to establish an endemic infection. Thus $R_{0}$ might be seen as a natural expression of the “receptivity” of a place to malaria because it is an intrinsic property of the area, relating to a state in which the parasite, immunity and control measures are absent. This use presents problems for the interpretation of “receptivity” in the pre-elimination period and the period following interruption of transmission when parasites may be present, interventions may remain in place and acquired immunity still remains in the population.

When transmission reducing interventions are in place, $R_{0}$ is a less relevant quantity as it only relates to a counter-factual scenario in which all interventions, parasites and immunity are removed from the population. In these situations a more accurate contemporaneous description of transmission potential is $R_{C}$, the controlled reproductive number. $R_{C}$ describes the transmission potential for an area where all parasites are removed along with acquired immunity, but other active interventions remain in place. As such $R_{C}$ is by definition lower than $R_{0}$, but still remains an expression of the initial maximum transmission that would be experienced in an area where parasites were newly introduced. Similarly to $R_{0}$, $R_{C}\ge 1$ correspond to situations in which an invading pathogen will establish endemicity, and values of $R_{C}<1$ correspond to situations in which an invading pathogen will fail to establish endemicity, and values of $0<R_{C}<1$ correspond to situations in which an invading pathogen might establish short chains of transmission that eventually end and fail to establish an endemic infection. In the context of this discussion, however, the $R_{C}$ values all correspond to maximum potential transmission as might occur considering human intervention. As such, $R_{C}$ could also be a natural proxy for “receptivity” with an added benefit that it would be more immediately relevant to situations in which countries had recently eliminated malaria but continued to maintain transmission reducing interventions.

Lastly, $R_{e}$, the effective reproductive number, which is not a measure of transmission potential but of actual contemporaneous transmission. As can be seen in Figure 1, $R_{e}$ varies with changes in prevalence of infection. When $R_{e}$ is $<1$ prevalence is declining, and when $R_{e}>1$ prevalence is increasing. After elimination it may not be measurable (if too few human cases occur either because of limited importation or too little subsequent transmission) but would be expected to approach the ceiling set by $R_{C}$ or $R_{0}$ as acquired immunity in the population wains. When $R_{e} = 1$ the system is at equilibrium. $R_{e}$ may not fully describe transmission “potential” where interventions and or immunity is absent, but is the easiest quantity to measure in the context of epidemiological investigation.

The reproductive number variants are challenging to utilize as proxy measures of receptivity. Both $R_{0}$ and $R_{C}$ refer to specific counter-factual situations in which full transmission potential is considered but in the absence of acquired immunity and interventions (for $R_{0}$; only the absence of acquired immunity for $R_{C}$). Neither can be directly measured, and their values are dependent on the model formulation used to estimate them. $R_{0}$ and $R_{C}$ can only be estimated based on an agreed mathematical model unless one accurately observes the initial stages of an epidemic in an area in which transmission has been absent for a long enough period for acquired immunity to have faded and specific transmission events can be enumerated. In which case it can be effectively estimated by counting secondary infections. The effective reproductive number $R_{e}$ can be similarly directly estimated in many situations at or near elimination, but only captures contemporaneous transmission rather than transmission potential and, unlike vectorial capacity, cannot be measured without cases of malaria.

Two recent studies in Eswatini, previously called Swaziland, and one in El Salvador attempted to assess receptivity to malaria transmission through the explicit use of human data and estimation of a reproductive number [23, 24, 33]. These studies illustrate both the challenges and potential of measuring receptivity through reproductive numbers. In the first published study by Churcher et al., the numbers of imported and local cases were used to estimate the $R_{e}$ at the national level for Eswatini [24]. This is a straightforward approach to measuring the current effective reproduction number which under some circumstances, post-elimination will reflect the $R_{C}$, see Fig. 1. One problem with this approach is that it estimates receptivity at the national scale (though it would in theory be possible to translate to smaller scales assuming sufficient importation), which is of little use to programmes for decision making on smaller scales. Perhaps more dangerously, geographic heterogeneity may lead to erroneous conclusions that transmission is indeed interrupted nationwide, when in fact it is only interrupted “on average”. It is simple and straightforward to extend the approach of Churcher et al. mathematically to any scale, but it requires that surveillance systems are able to accurately classify cases as local or imported to every geographic area of interest in the country, not only at the national level, a practice that is not typically undertaken, and is much more challenging than the national level importation classification process that countries use to inform policy and apply for WHO elimination certification.

The study by Reiner et al. describes the calculation of spatially (and temporally) explicit values for the effective reproduction number (though this is referred to as $R_{C}$ throughout, likely because the model implied in the Reiner et al. paper does not include immunity and as such little distinction between $R_{C}$ and $R_{e}$ is meaningful) [23]. A similar method is also used to estimate the importation of malaria and these two quantities are combined to produce spatially explicit maps of receptivity, rate of importation (formerly called vulnerability) and malariogenic potential (Malariogenic potential is the potential for malaria transmission in a given place and time arising from a combination of receptivity and risk or rate of importation (vulnerability)). The Reiner et al. study uses explicit spatial information and model based geo-statistics to evaluate the effective reproduction number at every location in Eswatini, thereby alleviating some of the challenges with the method of Churcher et al. but the reliance on $R_{e}$ (dubbed $R_{C}$ in Reiner et al.) means that this specific approach may not provide meaningful information about counter-factual scenarios for other places in which intervention coverage or immunity is removed. It also presents challenges for future use of the estimates in these locations when or if intervention coverage or population immunity changes. The study by Routledge et al. from El Salvador adopts a related approach though relying solely on temporal information about cases to infer transmission networks [33].

Because reproductive numbers can be estimated from data which are routinely collected by elimination programmes, it may be more useful for programs that need to develop stratification plans considering receptivity. The formalization of estimation requires statistical and mathematical knowledge and model assumptions, but these systems can be developed relatively inexpensively if the data to support the calculations is available. Since programmes have generally already made significant investments in routine data systems, much of the challenge and cost of data collection for measurement of “receptivity” through these methods have already been undertaken by programs.

### Other definitions of receptivity that have been quantitatively operationalized

The main alternative definition of “receptivity” which was identified through the literature review is based on historical estimates of parasite prevalence [5, 34, 35]. This application utilizes historical measurements of parasite prevalence, possibly modeled to extrapolate to areas without local data to summarize the history of malaria prevalence in a location. The maximum values over a relevant historical period are then taken to be the receptive risk of the area. Depending on model structure and data availability, secular trends and removal of interventions to create a counterfactual scenario. Historical estimates of *Pf*PR$_{2-10}$ or similar quantities are also potentially useful methods for assessing receptivity, but are subject to some important limitations including that (a) many areas of the world lack systematic surveys of parasite prevalence, (b) older prevalence studies may have been conducted prior to modern approaches to diagnosis and treatment and, (c) changes in ecology and other developments may have occurred which are not easily or accurately incorporated into models of malaria parasite prevalence and thus may lead to biased or inaccurate assessments of contemporary receptivity.

Measurement of “receptivity” using malaria prevalence data may also be possible with relatively little new program investment, where historical cross sectional data exists. One serious limitation is that these datasets are more likely to come from highly receptive areas than less receptive ones or areas which are no longer considered to be receptive, because cross sectional surveys tend to provide less value where the outcome of interest (malaria prevalence) is extremely rare and thus are often restricted to areas where malaria receptivity is comparably higher. Where programmes need to differentiate between areas with varied levels of receptivity, however, this data and approach could be very useful.

### Relationship between vectorial capacity and the reproductive numbers

Vectorial capacity and the basic reproductive number are related through the infectiousness of mosquitoes to humans (vector competence), humans to mosquitoes and the inclusion of the duration of infectiousness in humans. $R_{0}$ can be expressed in a classical formulation as per Eq. 2:

2 $$\begin{aligned} R_{0} = \frac{ma^{2}bc}{-ln(p)r}p^n \end{aligned}$$

where the newly introduced terms *b*, *c*, and *r* represent mosquito infectiousness to the human, human infectiousness to mosquitoes and the daily rate at which a human recovers from infection. $R_{C}$ can be constructed from the same inputs but considers the parameters under control rather than in a setting in which interventions to prevent malaria have not been applied.

The vectorial capacity in the Macdonald–Ross or other simple malaria models also correspond to the transmission potential in a simple SIS model for malaria in humans (transmission potential in these models is usually denoted as $\beta$ which is sometimes called the effective contact rate) and is related to $R_{C}$ and $R_{0}$ via the duration of infectiousness (or recovery rate). This simplification of transmission potential to focus only on human epidemiological factors can be estimated from readily available human data with only a time-series of numbers of cases identified and treated, and more than one measurement of community prevalence [36]. This allows the controlled reproductive number, $R_{C}$, to be estimated directly from human data on incidence and prevalence, conditional on an assumption about the probability per unit time of recovery from a malaria infection.

### *Plasmodium vivax*, relapsing and recrudescent malaria

In general, the areas considered to have any level of receptivity to *Plasmodium vivax* are believed to cover a wider geographic area than those with any level of receptivity to *Plasmodium falciparum* because the *P. vivax* parasite is capable of developing in the mosquito at lower ambient temperatures than *P. falciparum* [37]. This is mitigated to some extent by the widespread presence of Duffy blood group negativity ($FY^{*}B^{ES}$) in sub-Saharan Africa [38]. The presence of a dormant liver stage in *P. vivax* and *Plasmodium ovale* results in a complication to measuring receptivity to malaria based on human data. For these parasites, significant proportions of incident malaria cases may actually be the result of relapsing malaria arising from hypnozoites rather than directly related to recent transmission of parasites by mosquitoes. The treatment of these cases as locally acquired incident malaria infections results in an upward bias in the assessment of receptivity. This, while conservative for the determination of success in elimination [24], would likely result in higher resource use for programmes given the inflated need to respond more actively with vector control or treatment in areas misclassified as having higher than actual receptivity. In order for human data on malaria incidence to be used in estimation of receptivity to malaria in any location it will be important to consider classification of cases as due to relapse vs. newly acquired infection, as is recommended by the WHO, or an upwards bias in estimates of receptivity is likely. Recrudescent cases due to incomplete clearance could also introduce a similar upward bias in receptivity assessment if not fully differentiated from incident cases.

### Stratification of receptivity by parasite type

Overall the differences between *P. vivax* and *P. ovale* and the non-relapsing malaria parasites demonstrate that there is a need to estimate receptivity separately for each malaria parasite species anywhere that such estimates are required. Additionally, it is clear that there are differences in vector competence for different strains of parasites within and across species. For example some European malaria vectors, e.g. *Anopheles atroparvus*, are known to have poor competence or complete refractoriness for transmission of sub-Saharan African strains of *P. falciparum*, despite being highly competent vectors of Eurasian strains of *P. falciparum* [39–41]. Thus an estimate of receptivity for an area must consider at least the potential composition of parasite species and perhaps strain. This could be achieved by stratification of the assessment by mosquito species, parasite species and likely origin of imported parasites. To prevent the assessment of receptivity from requiring too many measurements it should consider only locally dominant vectors, parasites to which humans are susceptible locally and areas of origin from which migration is likely.

### Geographic scope and scale of receptivity assessment

Receptivity may vary dramatically over very small scales in many settings. Programmes are unlikely to be able to utilize very fine scale estimates for programmatic decisions and estimates made on too broad a scale (i.e. nationwide) are also likely to have limited utility. It seems then most relevant to make estimates of receptivity at a geographic scale aligned to that which is recommended for stratification by the WHO—the smallest administrative unit at which operational decisions can reasonably be made [4]. The geographic scope for receptivity measurement and classification should generally cover all areas of a country. Areas where no information is available could be assessed by extrapolation based on geo-statistical or other models or heuristically by comparison to areas with data until more direct measurements are available. In some countries it may be known, based on historical information or environmental attributes (e.g. altitude), that some areas may be completely non-receptive. Such areas should clearly be demarcated as operational responses to malaria cases in such areas will be very different than those in known receptive areas. Assessment of receptivity near borders may be particularly important in planning and assessing the appropriate intervention strategies to reduce, control or eliminate border malaria.

### Temporality and measurement of receptivity

Except for countries which long ago eliminated malaria and ceased vector control activities or which have had limited or no transmission without intervention for extended periods and whose populations can be expected to have no acquired immunity to malaria infection or disease, most countries will have no place where $R_{0}$ accurately reflects current transmission potential. The controlled reproductive number, $R_{C}$, is more likely to be reflective of transmission potential in these settings. Receptivity should be estimated using the most current data available, as the use of historical data requires ignoring or extrapolating to account for secular changes in factors such as climate or urbanization. Where a vectorial capacity approach is used, current data should also be employed. Though here care must also be taken to avoid over dependence on short term (or small scale) fluctuations in vector density affecting estimates.

Receptivity will be most useful to programs when it represents their current status rather than a historical counter factual. One appealing approach demonstrated in the work of Noor et al. is the use of historical data on maximum $PfPR_{2-10}$ as a proxy for transmission risk [34, 35]. While this approach is straightforward and appealing, it does have a risk of uncontrolled secular change resulting in biased estimates of receptivity. While historic trends have broadly been towards declining receptivity worldwide, there is no guarantee that this remains the case or is universally true. Thus the use of historical counter-factual may result in over- or under-estimation of receptivity.

### Qualitative approaches to receptivity estimation

In practice some malaria programs conduct qualitative or semi-quantitative assessments of receptivity utilizing bespoke instruments. In Malaysia, for instance, the assessment of the “receptivity” of an ecological area consists of scoring, on a nine point scale, mainly using entomological data. The assessment requires human landing catch, assessment of indoor and outdoor resting density, assessment of identifiable breeding sites for larval presence and distance to human habitations, assessment of parity rate and also identification of sporozoite or oöcyst positive mosquitoes and thus requires significant local entomological effort. The resulting items are combined with item specific weights to produce a receptivity index. These scores are then combined with a related index of vulnerability (importation risk) and used to calculate a joint index of malariogenic potential which guides programmatic response. Other similar approaches are practiced in a number of pre-elimination or prevention of re-establishment settings. In the PAHO region classification of areas purely qualitatively based on the presence or absence of vector species has also been recommended.

### Incorporation of parasite genetic information

Advances in genetic and genomic methods have created opportunities for genetic information from parasites to inform the assessment of receptivity in the field. While genetic information about parasites alone may not be sufficient to directly measure receptivity, such information could be used to improve the accuracy of the establishment of transmission chains and therefore contribute to estimation of receptivity through the effective reproductive number [23]. Such approaches have been used in epidemic monitoring of other human pathogens, but complexity of infection, high variability in parasite genomes and sexual reproduction make the use of genetic information in establishing transmission links more challenging than in other pathogens [42–44]. Additionally, genetic markers from parasites could potentially be used to differentiate local from imported infections, possibly enhancing the estimation of reproductive numbers through methods such as those proposed by Churcher et al. [24]. Finally in many cases multiplicity of infection, which can be measured by modern genetic methods, may also serve as a proxy for transmission intensity and thus for current receptivity, though evidence is stronger for associations on broad geographic scales than on small scales [45].

### Discussion

Reproductive numbers, vectorial capacity, and the use of historical/counterfactual $PfPR_{2-10}$ are suitable methods to assess the receptivity of local areas to malaria transmission and each has unique strengths and weaknesses. Recent advances in statistical methods including the application of model based geo-statistics allow for much finer grained mapping methods to differentiate receptivity on small scales. Use of a reproductive number approach requires human case data and these cases must be species specific and classified as local or imported (and relapsing in areas with relapsing malaria species present) with high accuracy and geographic specificity. Care should also be taken to ensure that recrudescent cases due to incomplete clearance are only counted once rather than double counted as new infections when repeated care seeking occurs due to the return of symptoms. Such cases can be common when the first line drug treatment has a substantial failure rate and can occur a considerable time (weeks or even months) after the primary treatment is applied.

Estimating vectorial capacity requires assessment of most or all of the parameters in Eq. 1. However, measurement of the human-biting rate during the transmission season may be all that is necessary in practice, since it has been argued that this is the main determinant of variability in the construct [20, 21]. Additionally it may be possible to utilize human immunological responses to *Anopheles* saliva as a proxy for biting rates [22].

Direct assessment of historical, pre-intervention $PfPR_{2-10}$ is obviously only possible where such historical prevalence data already exist. Alternatively, prevalence data in the presence of some interventions, or more recently acquired prevalence data, could be used to calculate counter-factual scenarios representing transmission potential or “receptivity” in the absence of intervention with appropriate statistical models. Such work has been conducted globally stretching as far back as the 1960s and most currently by the Malaria Atlas Project (MAP) [46, 47]. These data might serve as first pass estimate of current “receptivity” and counter-factual “receptivity.” In areas at or near elimination, prevalence is likely to be so low as to preclude the use of current prevalence data because of very small numbers of positive events, even in large surveys.

Despite the advances in methods available for receptivity assessment, many countries use methods that are qualitative or are based on indicies composed of measures that can be easily assessed by trained entomological and epidemiological surveillance teams during outbreak investigations or routinely as part of regular programme activities. These methods may also provide easy ways to assess receptivity which are implementable without specialized genetic, modelling or entomological capacity and may be suitable as well though none have been formally validated.

#### Minimum essential data

The minimum essential data necessary for estimation of the reproductive number in any form is a count of cases by month classified as local or imported (and relapsing or recrudescent) from an appropriate sized administrative area. Much better resolution assessment of receptivity can be made if case-based surveillance is conducted and local cases are geo-located to their most likely location of infection (usually the home, but potentially to a work place or other location). In the post-elimination period or prevention of re-establishment period, it is possible that the number of introduced cases (local cases with a direct epidemiological link to an imported case) is zero or nearly so. In such situations it may only be possible to estimate an upper limit of plausible $R_{C}$ values based on the absence of local cases and the frequency of imported cases (A. Ghani—*pers. comm.*). Furthermore in the absence of recent importation older classified case data might be used or extrapolations made from similar areas using heuristics or model based geo-statistics are also possible [23, 33].

Vectorial capacity requires a more extensive set of data, including locally measured human biting rate, parity (or parous rate) and human blood index. This info needs to be coupled with locally relevant estimates of the extrinsic incubation period which may be made with information only on local temperature/humidity derived from weather station or remote sensed data sets. Some components of vectorial capacity are likely more important in determining variability in “receptivity” than others and it may therefore be possible to create a measurement of “receptivity” using a more limited dataset [20]. Additionally, some components may be known historically and assumed to change little over time or space providing an approach to estimation of vectorial capacity which requires less intensive field work.

Historic $PfPR_{2-10}$ requires the availability of data from a pre-intervention period which can be localized to specific areas, or recent data on $PfPR_{2-10}$ with a substantial amount of information on confounding factors such as intervention coverage, local weather and climate, to allow for the construction of an appropriate counter factual prevalence scenario. As a first attempt at assessment of receptivity, maps of $PfPR_{2-10}$ or $R_{C}$ have been produced globally by MAP [46].

#### Definition and assessment

The practical use of “receptivity” as a construct in malariology suffers from conflicting and inconsistent definitions and lack of clear guidance on its measurement or assessment. In order to improve this situation, the WHO has adopted a clearer definition of “receptivity” and provides direct guidance on its assessment and use in programme decision-making [2]. Malaria elimination programmes, such as the Malaysia programme, have used receptivity as one component of an index for nationwide stratification, and often find data collected in the process useful in additional ways. Malaysia also uses entomological data collected during receptivity assessments to guide the choice of appropriate local vector control strategy [48]. Some countries, such as Eswatini, have used assessments of receptivity to determine where reactive case detection activities need to be focused [49, 50].

“Receptivity” itself is a construct, thus no formal “gold-standard” is available to evaluate assessment methods against. Despite this, comparative studies of assessment methods for “receptivity” have not been undertaken. A useful step would be to conduct comparison studies of “receptivity” using multiple methods within a single setting to determine whether the various methods proposed as acceptable here are consistent when compared, and whether one method might be preferable.

The assessment and measurement of “receptivity” through the use of a reproductive number, vectorial capacity or historic $PfPR_{2-10}$ are proposed as acceptable methods for receptivity assessment. In some cases, (such as the use of reproductive numbers) data collection could utilize only human case data, making assessment relatively low cost for programs which will collect such data anyway. One major limitation of human case based approaches to “receptivity” estimation is that the surveillance system must effectively capture nearly all cases. Without substantially complete data, all reproductive number estimates may be biased downwards. In elimination settings, programs must demonstrate surveillance capacity to capture nearly all malaria cases, if they intend to receive certification from WHO. As such, this concern may be less relevant in truly near elimination settings pursuing such certification. MAP has already produced maps at administrative level 2 of the controlled reproductive number estimates for *P. falciparum* globally (as well as $PfPR_{2-10}$ and *Pv*PR$_{1-99}$). While these estimates may still be at too broad an area for small scale program use in many settings, they also represent a publicly available assessment of receptivity for all endemic areas of the world [46].

Assessment of vectorial capacity has the longest history of use in the measurement of “receptivity.” While in theory vectorial capacity is an ideal measure of this construct, in practice the collection of the requisite components can be challenging, expensive and time consuming [20]. Additionally, the sampling of locations are rarely taken in a manner representative of the full landscape. Further measurement requires multiple measurement methods which make capturing covariance between components challenging as they cannot all be measured in the same mosquitoes and often are not even measured at the same times and in the same locations. Despite these challenges, there are relatively few examples of the use of statistical methods to extrapolate between measurements of vectorial capacity. It may also be possible to capture much of the variability in this measure through measurement of only some of its components [20]. While more research into the use of human immunological responses to *Anopheles* saliva is needed, such approaches might also be able to substitute for assessment of receptivity in areas where there is no or limited parasite presence [22].

In practice, many countries assess “receptivity” through other proxy methods. The methods used for these approaches generally focus on entomological observation or qualitative assessment of landscapes with an eye to ecological suitability for mosquito vector development. Neither the response of malaria programmes to malaria cases in receptive areas, nor the decision to maintain vector control strategies should be sensitive to small changes in receptivity. Since receptivity assessments will generally be used to stratify countries into a small number of classes (e.g. no receptivity, low receptivity, moderate receptivity, and high receptivity) for decision making purposes, such approaches might in fact be perfectly suitable.

### Conclusion

The current WHO definition of receptivity provides a good general framework for understanding the construct but requires additional guidance to help guide programs on how to measure and use this construct. Currently assessment of receptivity via the reproductive numbers provides the most intuitive stratification system and methods of assessment, which require only that human surveillance data are available. All elimination programs are already expected to collect such data and to classify cases in the manner that would allow this data to be used for receptivity assessment. Other assessment methods, namely those drawing on basic entomological surveillance or qualitative assessments of the landscape, are potentially useful, but many of the methods which are currently used have not been tested or validated against assessments based on vectorial capacity, historic $PfPR_{2-10}$ or the reproductive numbers. Programmes in the elimination or prevention of re-establishment stage should develop capacity in assessment of receptivity in order to ensure that they have reliable data on receptivity to use in planning and stratification.

## Data Availability

Not applicable.

## Appendix

### Glossary

#### Receptivity

Degree to which an ecosystem in a given area at a given time allows for the transmission of Plasmodium spp. from a human through a vector mosquito to another human. *Note: This concept reflects vectorial capacity, susceptibility of the human population to malaria infection, and the strength of the health system, including malaria interventions. Receptivity can be influenced by ecological and climatic factors.*

#### Reproductive number

The number of new infections expected to result from a single index infection.

#### Vectorial capacity

Number of new infections that the population of a given vector would induce per case per day at a given place and time, assuming that the human population is and remains fully susceptible to malaria.

#### Vector competence

For malaria, the ability of the mosquito to support completion of malaria parasite development after zygote formation and oöcyst formation, development and release of sporozoites that migrate to salivary glands, allowing transmission of viable sporozoites when the infective female mosquito feeds again *Note: Human malarias are transmitted exclusively by competent species of Anopheles mosquitoes; various plasmodia are transmitted by competent species of mosquitoes of the genera Aedes, Anopheles and Culex and other haematophagous Diptera.*

#### Model based geo-statistics

The application of parametric stochastic statistical models and likelihood-based methods of statistical inference to problems of continuous spatial (and possibly temporal) variation.
